# High-content CRISPR screening in tumor immunology

**DOI:** 10.3389/fimmu.2022.1041451

**Published:** 2022-11-21

**Authors:** Erin A. Holcomb, Ashley N. Pearson, Kassidy M. Jungles, Akshay Tate, Jadyn James, Long Jiang, Amanda K. Huber, Michael D. Green

**Affiliations:** ^1^ Graduate Program in Immunology, School of Medicine, University of Michigan, Ann Arbor, MI, United States; ^2^ Department of Radiation Oncology, School of Medicine, University of Michigan, Ann Arbor, MI, United States; ^3^ Department of Pharmacology, School of Medicine, University of Michigan, Ann Arbor, MI, United States; ^4^ Rogel Cancer Center, University of Michigan, Ann Arbor, MI, United States; ^5^ Institute of Health and Medicine, Hefei Comprehensive National Science Center, Hefei, China; ^6^ Department of Microbiology and Immunology, School of Medicine, University of Michigan, Ann Arbor, MI, United States; ^7^ Department of Radiation Oncology, Veterans Affairs Ann Arbor Healthcare System, Ann Arbor, MI, United States

**Keywords:** CRISPR screen, tumor immunology, cancer immunotherapy, transcriptomic readout, epigenetic readout, proteomic readout, genomic imaging

## Abstract

CRISPR screening is a powerful tool that links specific genetic alterations to corresponding phenotypes, thus allowing for high-throughput identification of novel gene functions. Pooled CRISPR screens have enabled discovery of innate and adaptive immune response regulators in the setting of viral infection and cancer. Emerging methods couple pooled CRISPR screens with parallel high-content readouts at the transcriptomic, epigenetic, proteomic, and optical levels. These approaches are illuminating cancer immune evasion mechanisms as well as nominating novel targets that augment T cell activation, increase T cell infiltration into tumors, and promote enhanced T cell cytotoxicity. This review details recent methodological advances in high-content CRISPR screens and highlights the impact this technology is having on tumor immunology.

## Introduction

Genetic perturbation provides biological insight. The development of genetic tools such as RNA interference (RNAi) have opened the door for genome-wide targeted perturbation enabling understanding of cancer cell and immune cell dependencies (1, 2). However, RNAi screening can be limited by incomplete protein knockdown and off-target effects (3–6). Discovery of CRISPR (Clustered Regularly Interspaced Short Palindromic Repeats)-associated Cas9 nuclease has revolutionized perturbation approaches by enabling knock out at the DNA level (7–10). Furthermore, the CRISPR-Cas9 system has enabled pooled genetic screening to be performed in an efficient manner compared to arrayed screens (11, 12). While arrayed screens use multiwell plates to separately target genes (13), pooled screens perturb a pool of genes in a single population of cells for increased throughput. Critical modules in performing a CRISPR screen include selection of an endonuclease system and guide library, screen phenotype selection, and hit evaluation. CRISPR-Cas9 screens have substantially advanced our understanding of innate and adaptive immune responses in the setting of infection and cancer (14–18). Furthermore, significant advances are being made in high-content phenotypic readouts at the transcriptomic, epigenetic, proteomic, and optical levels to increase resolution and overcome screening bottlenecks (19–22). This review highlights the critical modules necessary to perform a pooled CRISPR-Cas9 screen as well as discusses the recent development of high-content CRISPR screening systems. Furthermore, we detail how use of this technology is enabling discovery of novel therapeutic targets in the field of tumor immunology.

## CRISPR-Cas9 system

The Cas9 protein, derived from *Streptococcus pyogenes*, is the most commonly used Cas protein for CRISPR genome editing (23) due to its ability to introduce blunt double-stranded breaks (DSBs) in a cell’s genome at designated target sites. Error-prone non-homologous end joining (NHEJ) repair of these DSBs introduces a coding region frameshift through nucleotide insertion or deletion (indel) mutations which result in permanent gene silencing (7, 8). Cas9 is RNA-guided and can be programmed by guide RNAs (gRNA) to target specific genomic regions of interest, thus permitting robust loss-of-function studies (11–13). However, a limitation to the use of Cas proteins includes the inherent immunogenicity of this bacterial protein, which may reduce its efficacy when introduced *in vivo* (24).

More recently, the CRISPR system has been adapted for CRISPR interference (CRISPRi) and CRISPR activation (CRISPRa) studies (25–30). These methods utilize a catalytically inactivated Cas9 protein (dCas9) (7) that binds to genomic regions but is unable to cleave its target sequence for generation of DSBs. Fusion of dCas9 to activator or repressor domains results in targeted transcriptional activation or suppression at endogenous promotors, respectively. VP64 is a common transcriptional activator utilized for this system. Meanwhile, the Kruppel-associated box (KRAB) repressor domain can be coupled to Cas9 for transcriptional suppression (31). Thus, multiple CRISPR systems exist for genetic knockout, activation, or suppression studies and have become an appreciable tool for biological discovery.

### Designing a guide RNA library

The development of pooled single guide RNA (sgRNA) sequence libraries for CRISPR has enabled genome-wide functional screens which are scalable as well as cost- and time-efficient. When it comes to designing a pooled sgRNA library for a CRISPR screen, there are three general decisions to make. First, target genes must be selected. Smaller subpool libraries can be used to target a specific biological function, pathway, or gene family at greater depth. Alternatively, if there are no specific genes targets in mind, genome-wide library pools may be used to perturb any given gene in the genome. However, this approach requires more cells and increased sequencing cost and analysis time. Both subpool and genome-wide libraries are commonly used for *in vitro* and *ex vivo* screens; meanwhile, targeted subpool libraries are currently favored for *in vivo* immune cell screens rather than genome-wide libraries (22, 32, 33). The second step in this design process is to determine the library size, which can range from of 10^3^ to 10^5^ total sgRNA, with 2-10 redundant sgRNA targeting each gene. The specific numbers are arbitrary and depend on multiple factors. For instance, if cell count is limited or multiple models must be screened at once, one might opt to use 2-4 sgRNA per gene. On the other hand, if cost is not a concern, one can use 6-10 sgRNA per gene to observe weaker phenotypes with increased statistical confidence (34). Increased library size enhances screen resolution; for instance, the second-generation genome-wide Toronto KnockOut (TKO) library contains ~177 thousand sgRNA with up to 12 sgRNA per gene. This complex library has been used to screen for cancer cell fitness genes at increased resolution (35).

The final decision in this process is whether to use a custom or premade commercial sgRNA library. In a custom library, the screen can be tailored to genes of interest in a specific model. If conducting a CRISPR-knockout (KO) screen on protein-encoding genes, it is important to design custom sgRNA that target exon sequences. It is suggested to target transcripts within the first 50% of the coding sequence and also avoid proximity to the amino and carboxy terminus to prevent the use of alternative start codons or incomplete nullification, respectively (36). On the other hand, CRISPRi and CRISPRa screens often target promotors and regulatory elements. Although design tools are available to aid in this process, such as CrisprRGold, this process of guide design can be time-intensive (37). Premade commercial libraries alleviate this burden; however, they may not be validated or specific to a chosen model since they are intended to be simplified and generalizable. One approach to library selection is to conduct a primary screen with a commercial genome-wide library, then further investigate hit genes with a smaller custom library specific to a chosen model (34).

An important consideration when designing a sgRNA library is the potential for off-target effects. It has been shown that the Cas9 nuclease can still cleave a target DNA sequence even if it contains insertions, deletions, or mismatches to the guide RNA (38, 39). Methods have been developed to detect and quantify off-target cleavage by Cas9 including GUIDE-seq, HTGTS, BLESS, and Elevation (40–46). Titration of Cas9 and sgRNA concentration, as well as the inclusion of multiple redundant gRNAs per gene, can be implemented to minimize these off-target effects (38). Once library design is complete, the next step is to introduce the library into a target system.

### Introducing Cas9 and guide RNA into target cells

The CRISPR system can be delivered into a target cell by several routes. The most common method uses a retroviral or lentiviral (LV) vector, due to their large packaging size and permanent integration into the genome of dividing and non-dividing cells for long-term, robust expression of the CRISPR system (47). In comparison, adenovirus (AdV) and adeno-associated virus (AAV) are vectors that rarely integrate into the genome (48), which can limit off-target integration effects, although expression of the CRISPR system is less stable. Furthermore, the significantly reduced size of the AAV particle (~20 nm diameter) limits its ability to carry large insertions compared to the larger LV and AdV vectors (80-100 nm diameter) (49). Selection through antibiotic resistance reporter genes can improve the purity of transduced cells.

Delivery with viral vectors is more efficient than with lipid nanoparticles, which can encapsulate the Cas9 and sgRNA genetic material or sgRNA : Cas9 ribonucleoprotein (RNP) complexes. Viral vectors deliver the genetic material directly into the cytosol for immediate utilization, whereas lipid nanoparticles become endocytosed, and their contents may be degraded through the lysosomal pathway (49). As a result, lentiviral vectors serve as the most efficient and well-known means to stably introduce the CRISPR system into target cells. In the case of pooled CRISPR screens, a low multiplicity of infection (MOI) (~0.3) is often used to ensure that each cell is infected with only one virus, and thus only contains one perturbation (50–52). On the other hand, a high MOI may be used to induce multiple perturbations per cell for the study of genetic interactions (53, 54).

The Cas9 protein is quite large (~160 kDa), which may reduce viral packaging efficiency. Therefore, an important component to consider when gene editing is the delivery of Cas9 into target cells. If it is possible to generate Cas9-expressing cells, such as when screening a stable cell line, it is common to first transduce cells with a vector containing only Cas9, then transduce with a separate vector containing the gRNA. This method is known as a two-plasmid system (55, 56). However, it is difficult to stably express Cas9 in primary cells before gRNA introduction (57). Therefore, if screening animal primary cells, transgenic Cas9-expressing animals can be used. Alternatively, a single-plasmid system containing the genetic material of both Cas9 and sgRNA may be optimal when screening primary cells (56). In this setting, the challenges associated with transducing such a large protein can be circumvented through electroporation of Cas9 RNPs (58, 59). An additional advantage of electroporation is the elimination of genome integration induced by retroviral and lentiviral vectors, which may prevent the disruption of off-target genes and introduction of confounding variables.

Interestingly, delivery of Cas9 RNP complexes *via* electroporation has displayed higher efficiency than plasmid DNA, mRNA, or microinjection delivery (60) for inducing mutagenesis in mouse zygotes, induced pluripotent stem cells, and Jurkat T cells (61–63). Electroporation of Cas9 RNP complexes in an arrayed format has successfully edited the genomes of mouse primary CD8^+^ T cells and hematopoietic stem/progenitor cells (64, 65), as well as human primary CD4^+^ T cells, monocyte-derived DCs, and hematopoietic stem/progenitor cells (65–68). However, Cas9 RNP electroporation does not integrate the sgRNA into the genome, posing a problem for pooled screens whose readouts track integrated sgRNA. To enable pooled screening, lentiviral delivery of a sgRNA library can accompany Cas9 RNP electroporation to incorporate the sgRNA into the genome. For instance, a technique known as Guide Swap has successfully coupled RNP electroporation with pooled CRISPR screening (69). Guide Swap demonstrated that initial transduction with a pooled lentiviral gRNA library followed by electroporation of non-targeting RNPs induces efficient editing. This system has been validated in human primary CD4^+^ T cells and additionally introduced into hematopoietic stem and progenitor cells to uncover novel regulators of hematopoiesis including an actin-related protein (ACTR6) and corepressor complex (RCOR1) (69). Thus, the CRISPR-Cas9 system can be introduced into cells through electroporation alongside a lentiviral gRNA library to conduct pooled screens on primary immune cells.

One alternative to electroporation for delivery of Cas9 RNPs into target cells is the use of engineered virus-like particles (VLPs) (70–74). VLPs are pre-packaged with the Cas9 protein fused to a virion-targeted protein, and the presence of gRNA alongside these Cas9-VLPs allows for transient entry and editing by RNPs. This approach has been used to edit immune cell populations such as human monocyte and lymphocyte cell lines (72, 75, 76), primary mouse bone marrow cells (77), and primary human CD4^+^ T cells (72, 78) and monocyte-derived dendritic cells (71). VLPs have additionally been used for pooled screening of human induced pluripotent stem cell-derived macrophages (79). Notably, VLPs have successfully engineered chimeric antigen receptor (CAR)-expressing human CD4^+^ T cells without the use of *ex vivo* electroporation, highlighting their potential therapeutic application (73). Furthermore, addition of different glycoproteins or antibodies to VLP envelopes can mediate cellular tropism to induce selective editing of specific cell types and may be applied *in vivo* (73, 74, 80). For instance, addition of the CD4-tropic HIV-1 envelope glycoprotein enables selective editing of human CD4^+^ T cells, demonstrating targeted delivery of Cas9 RNPs (73). Engineered nanoparticles have likewise been used for *in vivo* delivery of RNPs to edit macrophages present within the liver and spleen of mice (81). Therefore, several approaches exist to introduce the CRISPR system into target cells for the purpose of screening, including primary immune cells.

## Conventional CRISPR screens in tumor immunology

**Figure 1:**
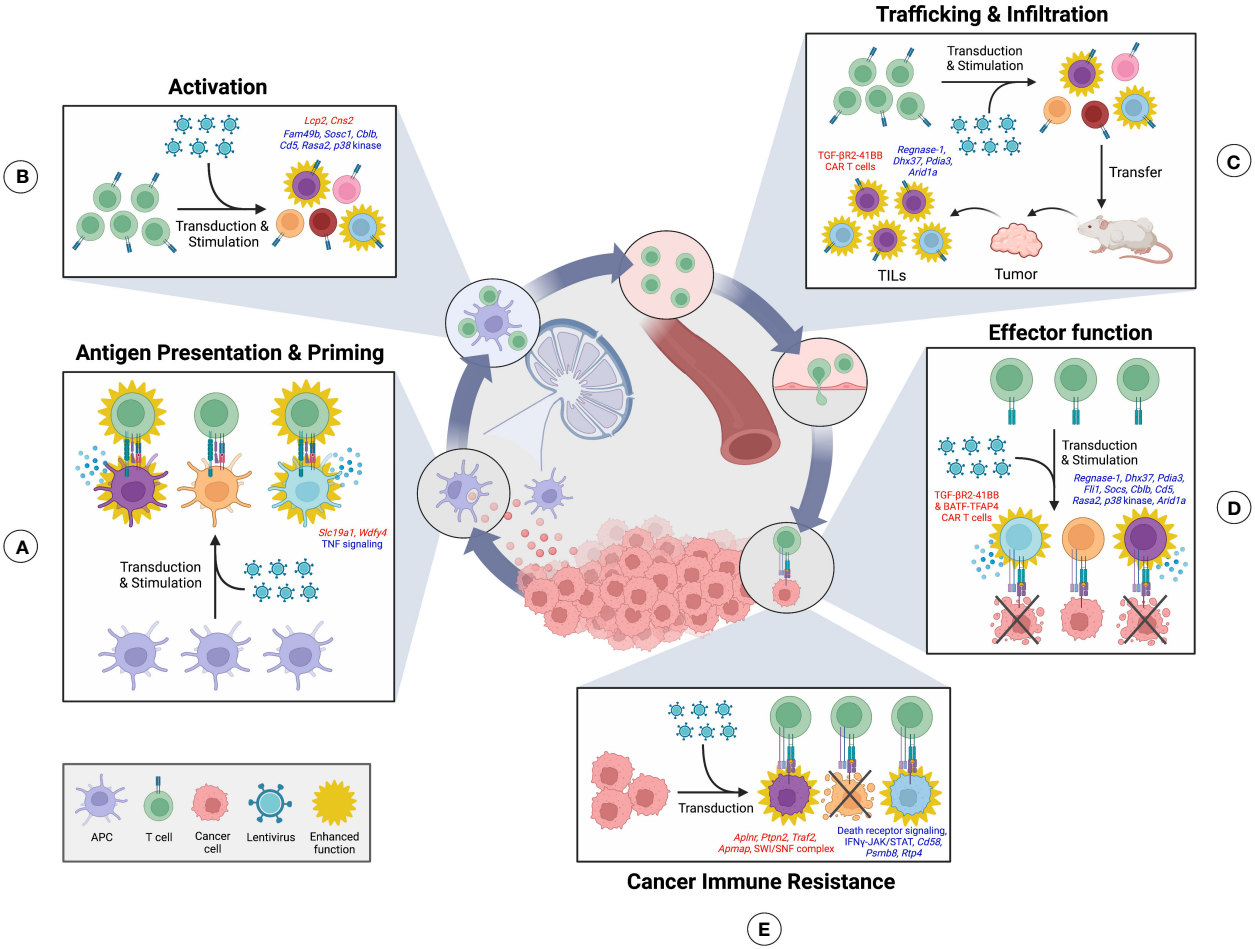
CRISPR screens can identify regulators of the Cancer-immunity cycle. The cancer-immunity cycle is a framework which describes the sequential generation of antitumor immune responses. High-throughput CRISPR screening can be used to screen cells in each step of this cycle to discover regulatory genes and their corresponding phenotypic effect. **(A)** In this cycle, tumor antigens are first released by cancer cells and sampled by antigen-presenting cells (APCs), such as dendritic cells, that may release cytokines in response to stimulation. APCs can then process and present captured antigens using major histocompatibility complex proteins on their surface. Trafficking of APCs to nearby lymph nodes allows for presentation of cancer antigens to naïve T cells for subsequent T cell activation. Screens on APCs can uncover genes regulating APC stimulation in response to tumor antigens and antigen presentation efficiency to T cells. **(B)** T cells that receive antigen stimulation become primed and activated towards a given tumor antigen. T cell screens can identify genes that mediate activation efficiency. **(C)** Primed T cells, such as cytotoxic T lymphocytes, can then egress from the lymph node, migrate through the blood, and infiltrate the tumor as tumor infiltrating lymphocytes (TILs). *In vivo* T cell screens can discover genes that promote TIL trafficking and infiltration. **(D)** Within the tumor, T cells are finally able to recognize their cognate cancer-specific antigen and induce tumor cell killing. T cell screens can identify genes that enhance tumor-killing activity. **(E)** Meanwhile, screening of tumor cells can uncover genes that mediate resistance to T cell killing. Positive regulators discovered at each step using CRISPR screens are shown in red, while negative regulators are shown in blue.

Conventional pooled CRISPR screens in numerous studies have uncovered genes that regulate the cancer immunity cycle (82) and may improve the antitumor response (83) (**Figure 1**). Readouts of these screens include cell survival, cell expansion, and phenotypic selection with fluorescence-activated cell sorting (FACS) based on fluorescent reporter or marker expression (84–86), followed by sequencing to identify enriched and depleted gRNAs. CRISPR screening of antigen-presenting cells (APCs) may detect genes regulating APC stimulation and antigen presentation efficiency to T cells (**Figure 1A**). For instance, a pooled screen in mouse primary bone marrow-derived dendritic cells (DCs) has identified factors controlling Tumor Necrosis Factor (TNF)α production in response to lipopolysaccharide (LPS) stimulation (86). These factors include the previously uncharacterized oligosaccharyltransferase (OST) complex and PAF transcription complex (86). Likewise, screens in immortalized and primary murine macrophages have similarly uncovered regulators of LPS-induced pathways (52, 87). For instance, *Mettl3*-mediated m^6^A RNA modification has been found to positively regulate LPS-induced macrophage activation to control tumor growth (87). Furthermore, autocrine TNF signaling has been surprisingly identified as a negative regulator of NF-κB (52). Overall, these screens used readouts of TNFα and NF-κB reporter expression (52, 86, 87). This screening approach may be applied in the future to investigate DC stimulation in response to tumor antigens. Finally, a pooled CRISPRi screen in the THP-1 human monocyte cell line using a cyclic dinucleotide (CDN)-inducible reporter system has revealed a CDN transporter, *SLC19A1*, as required for activation of the stimulator of interferon genes (STING) pathway (88). Importantly, induction of the STING pathway in antigen-presenting cells enhances antitumor responses (89).

Regulators of T cell activation have also been discovered by pooled CRISPR screens (16, 90, 91) (**Figure 1B**). Formation of the immunological synapse between T cells and APCs requires cytoskeletal remodeling to provide a proper signaling scaffold for T cell activation (92). A CRISPR screen in the immortalized Jurkat T cell line has uncovered *FAM49B* as a previously uncharacterized negative regulator of this cytoskeleton reorganization in response to TCR stimulation (90). Furthermore, known regulators of TCR signaling and proliferation have been confirmed in primary human CD8^+^ T cells (16), including the negative regulators *SOCS1*, *CBLB*, *CD5*, and *RASA2*, and positive regulator *LCP2*. CRISPRa screening has additionally been used to identify a novel stimulation-dependent IL-2Rα enhancer, *Cns2*, which influences T cell polarization. Deletion of this enhancer impaired regulatory T (Treg) development and favored differentiation into a pro-inflammatory T helper (Th) 17 state (91). Expression of common activation and proliferation features such as CD69, IL2Rα, and CSFE (16, 90, 91), have been used as a screen readout by these studies, followed by sorting and gRNA sequencing.

CD4^+^ T cells differentiate into distinct phenotypes in response to extracellular signals such as cytokines. Th1 cells are pro-inflammatory and enhance antitumor immunity. On the other hand, anti-inflammatory Th2 cells constrain Th1 response and Treg cells suppress immune functions to promote cancer progression (93). Pooled CRISPR screens in these T cell populations have revealed factors controlling their differentiation (94–98). An *in vivo* proliferation screen has identified *Socs1* as a negative regulator of CD4^+^ T cell proliferation, survival, and effector function indicative of a functional Th1 response. Notably, depletion of *Socs1* in CAR T cells enhanced antitumor immunity (94). Meanwhile, transcription factors *Pparg* and *Bhlhe40* identified by an *in vitro* screen have been revealed to mediate Th2 differentiation (95). Regulators of Foxp3 expression in Treg cells have also been pinpointed by *in vitro* screens. Positive regulators of Foxp3 include ubiquitin-specific peptidase 22 *(Usp22)* (a member of the SAGA complex) and the *Brd9*-containing ncBAF complex, while *Rnf20*, an E3 ubiquitin ligase, has been identified as a negative regulator (96, 97). Finally, mTORC1 signaling in Treg cells has been found to be promoted by *Sec31a* and the SWI/SNF complex and inhibited by the *Ccdc101*-containing SAGA complex (98. Inactivation of *Usp22*, *Brd9*, and *Ccdc101* enhanced antitumor immunity but also led to spontaneous inflammation in some cases (96–98). Thus, regulators of CD4^+^ T cell differentiation may be targeted to skew the balance towards a beneficial Th1 effector phenotype.

*In vivo* CRISPR screens have revealed mechanisms controlling tumor infiltration by CD8^+^ T cells in models of triple-negative breast cancer, glioblastoma, and melanoma (85, 99, 100) (**Figure 1C**). To do this, library-transduced CD8^+^ T cells were adoptively transferred into tumor-bearing mice and tumors were then sequenced. Depletion of factors identified by these screens have been shown to control CD8^+^ T cell effector function (**Figure 1D**) including ribonuclease *Regnase-1*, RNA helicase *Dhx37*, and ER-associated protein *Pdia3* (85, 99, 100), as well as the *Fli1* transcription factor identified by an *in vivo* LCMV Clone 13 infection screen (101). Modulation of effector function includes increased degranulation and tumor cell killing during *in vitro* co-culture, production of effector cytokines, expression of effector markers, as well as expression of inhibitory receptors (85, 99–101). Furthermore, adoptive transfer of CD8^+^ T cells deficient in these factors has been shown to reduce tumor growth and increase survival in murine tumor models. Notably, one of these studies has engineered regulator-depleted CAR T cells possessing *Pdia3* knockout to demonstrate potential clinical application (100).

Many T cell CRISPR screens involve *ex vivo* stimulation and transduction of isolated T cells; however, this process may disturb their homeostasis and differentiation. To address this issue, Sharpe et al. has developed an *in vivo* CRISPR-Cas9 delivery system, termed CHIME (CHimeric IMmune Editing) (102). CHIME consists of transducing bone marrow stem cells with a gRNA library *ex vivo*, followed by transfer into irradiated mice. In this manner, both innate and adaptive immune cell lineages can be rapidly screened *in vivo* without disrupting differentiation. This screening system has been applied in the context of LCMV Clone 13 infection (102) and may be implemented in future immunology screens to identify regulators of antitumor immunity.

Finally, multiple screens have elucidated mechanisms mediating resistance of cancer cells to CD8^+^ T cell killing and immune checkpoint blockade (ICB) (84, 103–106) (**Figure 1E**). To identify genes regulating resistance, cancer cells were first transduced with a gRNA library followed by either co-culture with CD8^+^ T cells *in vitro* or transplantation *in vivo* with or without ICB. Surviving cancer cells were then sequenced to identify genes that mediate resistance or susceptibility to the screening conditions. Screens in human melanoma cells have revealed mechanisms mediating susceptibility to *in vitro* CD8^+^ T cell killing, including an apelin receptor *(APLNR*) required for interferon gamma (IFNγ) susceptibility and components of the TNF pathway including *TRAF2* (84, 105). Alternatively, *in vitro* and *in vivo* screens have identified the SWI/SNF complex and a protein tyrosine phosphatase *Ptpn2* as mediators of resistance against the IFNγ response pathway, with inactivation of these factors enhancing the efficacy of ICB (103, 106). Moreover, an *in vitro* screen of CD19^+^ acute lymphoblastic leukemia cells co-cultured with CD19-targeted CAR T cells has found that deficiency in the death receptor apoptotic signaling pathway mediates cancer cell resistance and induces CAR T cell dysfunction (104). On the other hand, factors controlling macrophage antibody-dependent cellular phagocytosis (ADCP) have additionally been discovered by CRISPR-KO and CRISPRa screening of a B lymphocyte cell line (107). These factors include known ADCP regulators CD47 and CD20, as well as a previously undescribed enzyme adipocyte plasma membrane-associated protein *(APMAP*). In this study, screened immortalized B lymphocytes were co-cultured with stimulated macrophages and anti-CD20/CD47 antibodies to induce ADCP, then surviving cancer cells were sequenced. Loss of *APMAP* in cancer cells was shown to synergize with monoclonal antibody therapy and sensitize multiple tumor models to macrophage phagocytosis. Furthermore, a complementary genome-wide screen in macrophages revealed intercellular regulators of *APMAP*-deficient cancer cell uptake by macrophages, specifically the G-protein coupled receptor *GPR84* (107).

Conventional CRISPR screens have also been performed in an arrayed format to investigate regulators of the tumor-immunity cycle. For instance, arrayed CRISPR screening has been used to discover factors necessary for cross-presentation of tumor-antigens by conventional DC1s (cDC1s), most notably *Wdfy4* (15) (**Figure 1A**). In this study, primary murine CD8^+^ T cells were co-cultured *in vitro* with screened cDC1s, and T cell proliferation was measured as a readout of cross-presentation efficiency. Importantly, mice lacking *Wdfy4* were unable to suppress tumor growth (15). Furthermore, an arrayed screen in human monocyte-derived DCs (moDCs) has identified known regulators or Toll-like receptor (TLR) and MyD88 signaling in response to LPS from the human microbiome (67). Future screens may similarly investigate regulators of moDC signaling in response to tumor antigens. Finally, arrayed screening has revealed *p38* kinase as a regulator of CD8^+^ T cell activation (**Figure 1B**). Inhibition of *p38* in CD8^+^ T cells resulted in increased cell expansion and expression of the memory marker CD62L, as well as reduced ROS and DNA damage. Furthermore, CAR T cells depleted in *p38* kinase using a small molecule *p38* inhibitor exhibited enhanced effector function, shown by increased IFNγ production and cytolytic activity *ex vivo* (64) (**Figure 1D**). Thus, pooled and arrayed CRISPR screening is a tremendous tool used to uncover key regulators of the cancer-immunity cycle that may potentially be targeted in a clinical setting to improve therapeutic response.

## Coupling CRISPR screens to transcriptomic readouts

### Barcoded systems

**Figure 2:**
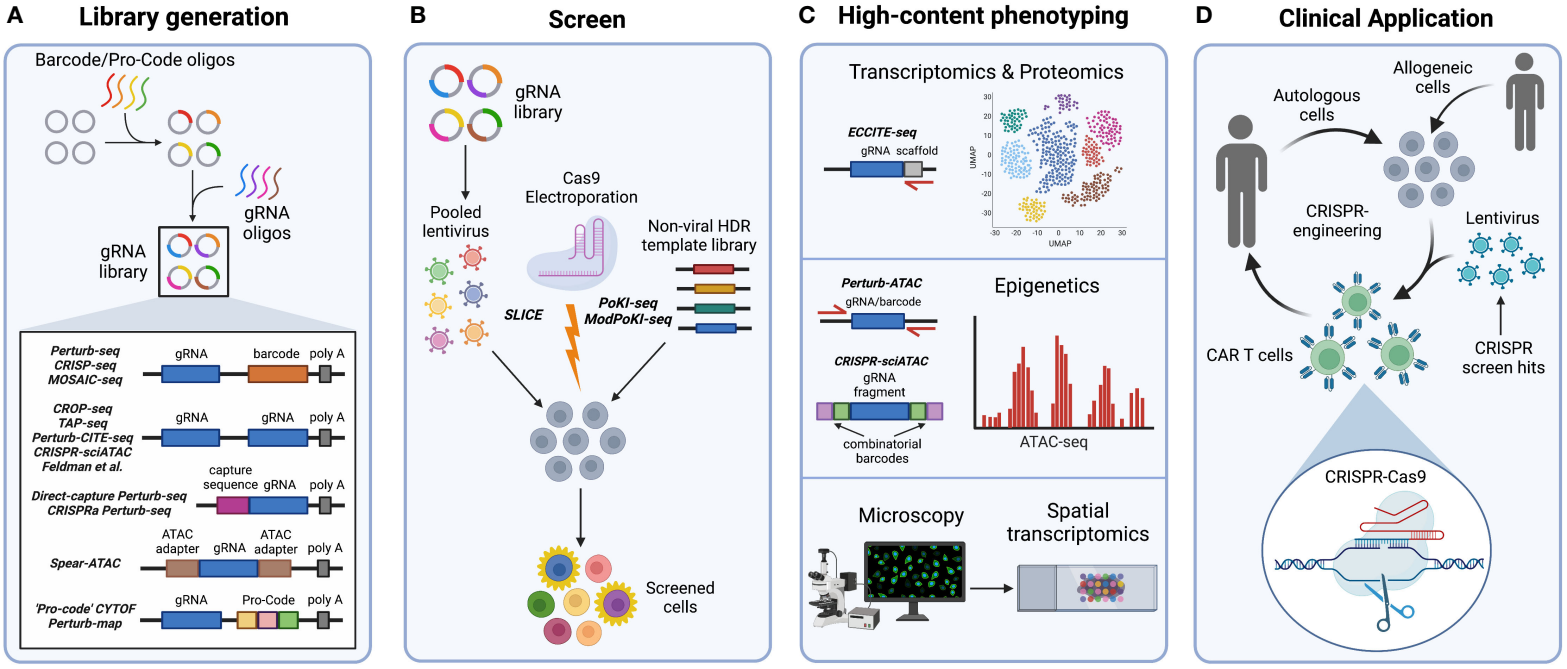
Steps in high-content CRISPR screening. **(A)** For systems that use barcodes or Pro-Codes, these oligonucleotides are first cloned into a viral vector, followed by guide RNA (gRNA) oligonucleotides to create unique paired gRNA-barcode/Pro-Code constructs that are recognized during sequencing. Meanwhile, systems that use the non-barcoded CROP-seq vector contain gRNA sequences that are directly sequenced without the need for barcodes. Furthermore, Direct-capture Perturb-seq and CRISPRa Perturb-seq vectors contain capture sequences within gRNA scaffold regions that are recognized during sequencing. In Spear-ATAC vectors, adapters used in ATAC-seq flank the gRNA sequence for direct amplification from the genomic DNA during ATAC-seq. **(B)** Introduction of the CRISPR-Cas9 system into cells for pooled content-rich screening can occur in multiple ways. Pooled vectors can be used to generate lentivirus, which then transduce and integrate into target cell genomes in the presence of Cas9. If Cas9 is not simultaneously transduced or expressed by target cells, SLICE (sgRNA lentiviral infection with Cas9 protein or electroporation) may be employed to introduce Cas9. Furthermore, the PoKI-seq system can be used for pooled knockin screening by simultaneously electroporating a gRNA : Cas9 ribonucleoprotein complex with a non-viral barcoded homology directed repair template library. **(C)** Phenotypic readouts of high-content CRISPR screens include transcriptomic, proteomic, epigenetic, and microscopy data. ECCITE-seq specifically adds gRNA scaffold-targeted primers to detect the gRNA during scRNA-seq. Perturb-ATAC uses pooled primers to target flanking regions of the barcode or gRNA during ATAC-seq. Meanwhile, in the CRISPR-sciATAC system, gRNA and ATAC fragments are labeled with combinatorial barcodes for detection during ATAC-seq. Finally, optical readouts include imaging followed by spatial transcriptomics. **(D)** The CRISPR-Cas9 system is a significant tool for clinical advancement. CRISPR-Cas9 editing may be used to target genes identified by immune cell CRISPR screens controlling antitumor functions. Autologous or allogeneic engineered cells, such as chimeric antigen receptor (CAR) T cells, may then be adoptively transferred into patients.

As previously mentioned, conventional pooled CRISPR screen readouts are unable to distinguish complex phenotypes that may require further deconvolution, which can be time-consuming. To address this issue, multiple groups have developed barcoded systems for pooled high-content CRISPR-Cas9 phenotyping (**Table 1**). The first of these systems have been termed Perturb-seq, CRISP-seq, and Mosaic-seq (51, 54, 108, 109) and couple CRISPR screening to a high-content single-cell RNA sequencing (scRNA-seq) readout using a “barcoded” vector library (**Figure 2**). To generate this library, barcode oligonucleotides are first cloned into a lentiviral vector near the 3’ polyadenylation (poly-A) tail. Close proximity of the barcode to the poly-A tail ensures detection by microfluidic scRNA-seq. After barcode cloning, gRNA oligonucleotides are further cloned into the vector and sanger sequencing is performed to pair each gRNA to its unique barcode within a single construct. As a result, cells transduced with this barcoded vector will be labeled with a barcode tied to the specific gRNA-induced genetic perturbation. This barcode identifies the gRNA, or combinations of gRNA, that infect each single cell during scRNA-seq analysis (51, 54, 108, 109).

**Table 1:** High-content CRISPR screening systems.

Readout	Description	Model	System studied	Reference
**Transcriptomic readout**				
*Barcoded systems*				
Perturb-seq	gRNA-specific barcodes proximal to poly-A tail are recognized during scRNA-seq	Murine BMDCs, Human K562 cells *in vitro*	LPS stimulation response, Transcription factor functions, Cell fitness	(54)
		Human K562 cells *in vitro*	Unfolded protein response	(51)
CRISP-seq		Murine BMDCs *in vitro* and *in vivo*	Monocyte lineage differentiation and LPS-induced inflammatory response	(108)
Mosaic-seq		Human K562 cells *in vitro*	Enhancer target genes, Combinatorial enhancer activity	(109)
PoKI-seq	‘Pooled knockin sequencing’ Electroporation of RNP with a non-viral barcoded homology-directed repair template library coupled to scRNA-seq	Primary human T cells *in vivo*	Engineer and phenotype CAR T cells	(32)
ModPoKI-seq	‘Modular pooled knockin screening’ Modified PoKI-seq to reduce template switching	Primary human T cells *in vitro* and *in vivo*	Engineer and phenotype CAR T cells	(110)
*Non-barcoded systems*				
CROP-seq	gRNA is proximal to poly-A tail for direct sequencing during scRNA-seq	Jurkat T cells *in vitro*	T cell receptor signaling	(19)
	CROP-seq paired with SLICE	Primary human T cells *in vitro*	T cell stimulation regulators	(16)
Direct-capture Perturb-seq	gRNA-specific primers bind to capture sequences in gRNA constant regions during scRNA-seq	Human K562 cells *in vitro*	Genetic interactions between cholesterol biosynthesis and DNA repair genes	(111)
		Human K562 cells, Human RPE1 cells *in vitro*	Mitochondrial stress response, ribosome biogenesis, integrator complex, erythroid and myeloid differentiation, and aneuploidy	(112)
		Murine TILs *in vivo*	Chromatin remodeling complexes in T cell exhaustion	(33)
CRISPRa Perturb-seq	Capture sequences in gRNA scaffold regions are recognized during scRNA-seq	Primary human T cells *in vitro*	Stimulation-response cytokine regulators	(113)
TAP-seq	‘Targeted Perturb-seq’ Gene-specific primers amplify transcripts of interest during CROP-seq	Human K562 cells *in vitro*	Enhancer-target gene pairs	(114)
**Epigenetic readout**				
Perturb-ATAC	Pooled primers target flanking regions of barcode or gRNA during ATAC-seq; Micro-well fluidics platform	Human GM12878 lymphoblastic cells *in vitro*	Nucleosome structure alterations	(115)
CRISPR-sciATAC	gRNA and ATAC fragments are labeled with combinatorial barcodes for detection during ATAC-seq; CROP-seq vector; Micro-well-based	Human K562 cells *in vitro*	Chromatin accessibility	(116)
Spear-ATAC	Pre-integrated adaptors flank gRNA for direct amplification from genomic DNA during scATAC-seq; Droplet-based	Human K562 cells, GM12878 lymphoblastic cells, MCF7 breast cancer cells *in vitro*	Epigenetic alterations to transcription factor binding sites	(20)
**Proteomic readout**				
ECCITE-seq	‘Expanded CRISPR-compatible CITE-seq’ gRNA scaffold-specific primers detect gRNA during scRNA-seq	Human K562 cells *in vitro*	Small sub-pool validation screen targeting known cell surface markers and intracellular signaling molecules	(117)
		THP-1 monocyte leukemia cells *in vitro*	Transcriptional and post-transcriptional regulators of PD-L1 expression	(118)
		Human cutaneous T-cell lymphoma samples *in vitro*	Malignancy of different T cell clonotypes, Characteristics of skin vs blood microenvironment	(119)
Perturb-CITE-seq	CROP-seq coupled with CITE-seq	Patient-derived melanoma cells *in vitro*	Mechanisms of ICI resistance	(21)
‘Pro-code’ CYTOF	Protein-encoding barcodes (Pro-Codes) couple CRISPR screens with mass cytometry	Breast cancer 4T1 cells *in vitro*	Regulators of breast cancer immune evasion	(120)
**Optical readout**				
Feldman et al.	Barcoded or non-barcoded CRISPR screens coupled with imaging	HeLa cells *in vitro*	Regulators of NF-kB signaling	(121)
Perturb-map	Protein-encoding barcodes (Pro-Codes) couple CRISPR screens with imaging and transcriptomics	Lung adenocarcinoma cells *in vivo*	Regulators of TME	(22)

Validation and application of the first high-content transcriptomic CRISPR screening systems has been conducted in both primary immune cells and cell lines and may be translated to a tumor immunology setting in the future. For instance, Perturb-seq screening with a pooled library targeting transcription factors has uncovered regulators of the LPS-stimulation response in murine bone-marrow-derived dendritic cells (BMDCs) (54). Importantly, coupled scRNA-seq analysis allows for classification of distinct transcription factor modules based upon individual perturbation effects on the transcriptome. Furthermore, identification of gene programs altered by these perturbations are indicative of differentiating cell states. For instance, a transcription factor module containing *Cebpb, Rela*, and *JunB* perturbations was shown to downregulate the LPS response gene program in BMDCs (54). Similarly, CRISP-seq has been performed on murine BMDCs with a pooled library targeting known transcription factors upregulated by LPS stimulation (108). This study uncovered the effect of these perturbations on monocyte lineage differentiation and the LPS-induced inflammatory response. Moreover, an *in vivo* CRISP-seq screen of hematopoietic progenitor cells in mice injected with LPS was performed to confirm *in vitro* results. Results of this study revealed that *Cebpb* promotes myeloid differentiation towards a monocytic state; meanwhile, *Irf8* promotes DC development. Furthermore, deletion of *Rela* and *Stat2* results in reduced expression of inflammatory and antiviral pathways, respectively. Going forward, this high-content screening approach may similarly be used to identify gene modules and programs mediating DC stimulation in response to tumor antigens (**Figure 1A**), as well as immune cell differentiation *in vivo*.

The original high-content transcriptomic screening systems have also been implemented in immortalized chronic myeloid leukemia K562 cells. Perturb-seq screening with a pooled library targeting cell-cycle regulators has characterized perturbation effects on cancer cell fitness by linking each perturbation to resulting cell-cycle phase gene signatures and fitness-related gene programs (54). Similarly, Mosaic-seq has applied CRISPRi to screen K562 cells with a pooled library targeting known enhancers. In this screen, coupled transcriptome profiling has identified enhancer target genes whose expression was altered in response to KRAB-mediated enhancer repression. Distinct constituents within super-enhancer regions were shown to control the expression of a leukemic oncogene, *PIM1* (109). This study additionally performed Mosaic-seq using a high MOI to investigate the effect of combinatorial perturbations on enhancer function. As a result, dual repression of certain enhancer constituents was shown to inhibit *PIM1* expression. Thus, pooled CRISPR screen studies have utilized transcriptome-coupled approaches to detect and characterize factors that mediate cancer cell fitness and oncogenic potential. In the future, high-content screens in tumor cells may alternatively reveal regulators of cancer immune evasion (**Figure 1E**).

Barcoded high-content transcriptomic screening has also been applied in human T cells using pooled knockin sequencing (PoKI-seq) (32) **(** **Figure 2B**), which consists of simultaneous electroporation of a gRNA : Cas9 RNP with a non-viral barcoded homology directed repair (HDR) template library followed by transcriptome sequencing. PoKI-seq has been used to engineer and phenotype human T cells expressing chimeric receptors inserted within the endogenous T cell receptor α constant *(TRAC)* locus. For instance, TGF-βR2-derived CAR T cells expressing an extracellular TGFβ synthetic receptor linked to intracellular stimulatory domains can convert inhibitory stimuli into stimulatory signals in the presence of TGFβ. PoKI-seq revealed that TGF-βR2-derived CAR T cells stimulated with TGFβ are similar in cell state to control cells stimulated without TGFβ, indicated by expression of genes related to proliferation and cytotoxicity (32). Moreover, *in vivo* PoKI-seq screening of engineered CAR T cells in human melanoma tumor-bearing mice has shown increased accumulation of TGF-βR2-derived tumor-infiltrating lymphocytes (TILs) within the tumor (**Figure 1C**). Transcriptome analysis further revealed that TGF-βR2-41BB TILs exhibit an effector cell state with increased expression of IL-2 and IFNγ, resulting in enhanced tumor clearance *in vivo* (32) **(** **Figure 1D** **).**

More recently, Modular Pooled Knockin screening coupled with transcriptome sequencing (ModPoKI-seq) has modified PoKI-seq to reduce template switching (110). To achieve this, barcoded adaptors are included on both the 5’ and 3’ ends of the knockin gene, resulting in increased proximity of the barcode to the gRNA (**Figure 2B**). This system allows for flexible perturbation identification at the DNA or mRNA level. ModPoKI-seq has been used to assess transcriptomic signatures of repeatedly stimulated CAR T cells possessing transcription factor knockin constructs within the *TRAC* locus. For instance, CAR T cells with BATF, BATF3, and TFAP4 knockins are enriched within a proliferation-associated cluster, indicating improved persistence and function even in the presence of repeated stimulation. Notably, dual BATF-TFAP4 expressing CAR T cells display enhanced antitumor function *in vitro* and *in vivo* (110) **(** **Figure 1D**). Therefore, high-content screenings systems such as PoKI-seq and ModPoKI-seq can be used to engineer and phenotype CAR T cells in an efficient manner.

Overall, these paramount approaches illustrate the potential application of barcoded pooled CRISPR systems to uncover complex phenotypes when coupled to scRNA-seq, as compared to conventional CRISPR screening. This emerging approach is promising for the field of tumor immunology to identify genes affecting immune cell antitumor function in the future (**Figure 2** **).**

### Non-barcoded systems

Despite the influential innovation of barcoded vectors such as Perturb-seq, CRISP-seq, and Mosaic-seq (51, 108, 109), recombination of gRNA and barcode during pooled lentiviral packaging presents a challenge when matching barcodes to corresponding gRNAs (122, 123). To circumvent gRNA-barcode recombination, non-barcoded systems have been developed for direct detection of gRNAs (**Table 1**). One system known as CROP-seq (19) inserts the gRNA cassette next to the 3’ poly-A tail (**Figure 2A**). As a result, the gRNA is included in the poly-adenylated transcripts detected by scRNA-seq instead of a separate barcode. CROP-seq pooled screening has been validated in Jurkat T cells by generating T cell activation signatures and identifying known genes important for T cell receptor signaling, such as *LCK* and *ZAP70* (19) **(** **Figure 1B**). CROP-seq has also been applied with SLICE (sgRNA lentiviral infection with Cas9 protein electroporation) **(** **Figure 2B** **)** to phenotype primary human T cells screened with a library targeting stimulation response regulators (16). Unsurprisingly, perturbation of negative proliferation regulators such as *SOCS1*, *CBLB*, *CD5*, and *RASA2* resulted in an enriched transcriptional program associated with cell-cycle and effector genes, as well as increased tumor cell killing *in vitro* (**Figures 1B-D**). Thus, CROP-seq has displayed appreciable potential for future studies involving primary immune cell pooled CRISPR screening with high-content readout.

A similar technique, known as direct-capture Perturb-seq, also directly sequences gRNA for identification in single-cell transcriptomes (111). To do this, direct-capture Perturb-seq introduces guide-specific primers that bind to capture sequences within gRNA constant regions during scRNA-seq (**Figure 2A** **).** Importantly, direct-capture Perturb-seq can detect different gRNA within a single cell. Thus, unlike CROP-seq, direct-capture Perturb-seq can be used in combinatorial pooled CRISPR-scRNA-seq screens that use libraries containing multiple gRNA for discovery of epistatic interactions. This approach was initially implemented with CRISPRi to identify DNA repair genes in human K562 cells (111), and has additionally been applied to a genome-wide combinatorial CRISPRi screen of K562 and retinal pigment epithelial (RPE1) cell lines (112). Using transcriptional profiles, novel regulators of the mitochondrial stress response, erythroid and myeloid differentiation, and aneuploidy were revealed (112). In the context of immune cells, *in vivo* direct-capture Perturb-seq has identified INO80 and cBAF chromatin remodeling complexes as regulators of CD8^+^ T cell exhaustion (33). This study shows that loss of *Arid1a* in murine TILs, a member of the cBAF complex, reduces exhaustion-associated genes and instead promotes an effector phenotype to enhance antitumor immunity following adoptive cell transfer (**Figure 1D**). More recently, Schmidt et al. has also developed a similar platform for direct capture of gRNA by incorporating a capture sequence in the gRNA scaffold region. This system, referred to as “CRISPRa Perturb-seq,” (**Figure 2A**) has been used to characterize pooled CRISPRa screen hits of stimulation-response cytokine regulators in primary human T cells (113) **(** **Figure 1B**). Coupling of CRISPRa to scRNA-seq has enabled analysis of cytokine expression, activation gene signatures, and cell states that are altered in response to individual perturbations. As a result, genes controlling NF-κB signaling have been identified, such as 4-1BB, OX40, CD27, and CD40. Therefore, direct-capture Perturb-seq may be implemented in the future to characterize regulators of immune cell function.

High cost and insufficient sensitivity for low expression genes remain shortcomings of single-cell sequencing screens. This has led to the development of targeted Perturb-seq (TAP-seq) (114) **(** **Figure 2A**) which combines the CROP-seq vector with gene-specific primers to amplify transcripts of interest following the reverse transcription step of scRNA-seq. This technique has been applied with CRISPRi to phenotype human K562 cells for enhancer-target gene pairs using a pooled library targeting active enhancers, then selectively detecting the resulting expression of nearby genes. This study revealed that the majority of enhancers are located proximal to their target genes, and that strong enhancer-target pairs contain higher levels of active chromatin marks (114). In the future, TAP-seq may be implemented in high-content CRISPR screen studies to detect rare gene targets in immune cell subsets at a more economical cost.

In summary, CROP-seq, direct-capture Perturb-seq, and TAP-seq methods exemplify direct gRNA capture as a means to identify CRISPR perturbations from scRNA seq data, as opposed to the use of barcoded gRNA vectors. These methods alleviate gRNA-barcode recombination to ensure accurate barcode assignment and may be employed in immune cell screens to generate high-content phenotypes.

## Coupling CRISPR screens to epigenetic readouts

Multiple techniques have been developed that couple CRISPR screens to epigenetic analyses (**Table 1**). Although these systems originally investigated contexts not pertaining to tumor immunology, future studies could use these methods to probe for epigenetic regulators of anti-tumor immunity in primary immune cells. For instance, CRISPRi and CRISPR-KO screens performed in an arrayed format have been linked with epigenetic data using Perturb-ATAC (115). Perturb-ATAC builds upon ATAC-seq (Assay for Transposase-Accessible Chromatin with sequencing), a method that implements the hyperactive Tn5 transposase with DNA sequencing to assess epigenetic profiling (124). To perform Perturb-ATAC, individual cells are captured on an integrated fluidic circuit and ATAC-seq is performed following pooled screening. The addition of pooled primers that target regions flanking the barcode or gRNA allows for simultaneous amplification of both ATAC-seq fragments and the barcode or gRNA linking the epigenetic data with the CRISPR-induced genetic perturbation (**Figure 2C**). Arrayed CRISPRi screening with Perturb-ATAC has been implicated in human immortalized B lymphoblasts to identify factors that induce nucleosome structure alterations, such as the chromatin regulator *DNMT3A* (115).

However, a limitation to Perturb-ATAC is that it is performed in an arrayed format and requires a fluidics platform which can only capture ninety-six cells. More recently, development of CRISPR-sciATAC, which uses the CROP-seq vector, has eliminated the need for a fluidics platform and instead implements 96 barcoded transposases for combinatorial barcoding of ATAC and gRNA fragments in 96-well plates (116) (**Figures 2A-C**). Pooled CRISPR-sciATAC screening has been used to screen chromatin-related genes in human myelogenous leukemia K562 cells. Linkage of specific gene perturbations to ATAC-seq data reveals changes in chromatin accessibility. For instance, loss of a SWI/SNF complex component, *ARID1A*, results in reduced accessibility at JUN and FOS transcription factor binding sites (116).

Furthermore, Pierce et al. has also increased throughput capacity by developing Spear-ATAC (Single-cell perturbations with an accessibility read-out using scATAC-seq) (20), derived from scATAC-seq (125). Since scATAC-seq is droplet-based, it allows for higher throughput and compatibility with pooled screens. In Spear-ATAC-seq, to detect each perturbation, adapters used in ATAC-seq are also inserted around the gRNA sequence (**Figure 2A**). Therefore, both ATAC-seq fragments and the gRNA are directly amplified from the genomic DNA, as compared to typical gRNA detection from RNA transcripts. Consequently, each gRNA perturbation can be easily paired to epigenetic data without the use of a barcoded vector system. Spear-ATAC coupled with pooled CRISPRi screening has been used to phenotype human K562 leukemia and MCF7 breast cancer cell lines, as well as GM12878 B lymphoblasts (20). By perturbing key transcription factors, Spear-ATAC has identified epigenetic alterations to transcription factor binding sites in K562 cells with increased throughput and reduced cost as compared to Perturb-ATAC (115). For instance, *GATA1* deletion resulted in increased accessibility of hematopoietic transcription factor binging regions such as *RUNX*, *SPI1*, and *IRF1* (20). Hence, these systems may be applied in future high-content pooled CRISPR screens to reveal potential epigenetic regulation relevant to antitumor immunity in various immune cell types.

## Coupling CRISPR screens to proteomic readouts

Proteomic data can be coupled to pooled CRISPR screens (**Table 1**), and this technology has exhibited promise in the field of tumor immunology. ECCITE-seq (Expanded CRISPR-compatible cellular indexing of transcriptomes and epitopes by sequencing) has been adapted from CITE-seq for the purpose of pooled CRISPR screening (117). CITE-seq functions to pair single-cell transcriptome data with protein epitope and TCR clonotype data using barcoded oligonucleotide tags conjugated to protein-specific antibodies that are simultaneously detected during scRNA-seq (126). ECCITE-seq builds off this concept by introducing an additional gRNA scaffold-specific primer to the scRNA-seq reverse transcription reaction (**Figure 2C**). Therefore, the gRNA sequence is directly captured and allows for linkage of transcriptomic, proteomic, and TCR clonotype data to gRNA-specific perturbations introduced during pooled CRISPR screens (117). ECCITE-seq was initially shown to be compatible with small-scale CRISPR screening in K562 cells using a small sub-pool library targeting known cell surface markers and intracellular signaling molecules (117). Recently, ECCITE-seq has identified both transcriptional and post-transcriptional regulators of PD-L1 expression in human monocyte leukemia THP-1 cells. Results from this study illustrate that PD-L1 upregulation following interferon IFNγ signaling is mediated by *NRF2* and *KEAP1* (118). Additionally, ECCITE-seq has been implemented in human cutaneous T-cell lymphoma samples to reveal transcriptional profiles indicative of malignancy in different T cell clonotypes, as well as transcriptional and proteomic differences in the skin microenvironment of CTCL patients compared to the blood (119).

More recently, Frangieh et al. has developed Perturb-CITE-seq for large-scale pooled screens by combining a modified CROP-seq vector with CITE-seq, as well as a computational framework for analysis of the perturbation data (21) (**Figure 2A**). Notably, Perturb-CITE-seq has been used to screen patient-derived melanoma cells with a library consisting of genes previously associated with ICI resistance. Melanoma cells are cultured with autologous TILs to identify known mechanisms of ICI resistance (**Figure 1E**), such as impaired IFNγ-JAK/STAT and antigen presentation pathways, as well as novel mechanisms such as *CD58* downregulation. Importantly, simultaneous scRNA-seq and CITE-seq phenotyping employed by Perturb-CITE-seq has revealed perturbation-induced effects at both the RNA and protein level in this model system.

Protein-level phenotyping of pooled CRISPR screens has also been achieved through high-throughput CyTOF mass cytometry (120). To do this, protein-encoding barcodes (termed Pro-Codes) are generated consisting of triplet combinations of linear epitopes that are detectable by known antibodies. These Pro-Code sequences are then paired with gRNA sequences within the lentiviral vector (**Figure 2A**). Following transduction, metal-conjugated antibodies specific to the linear epitopes detect each Pro-Code reporter by CyTOF mass cytometry to report the corresponding genetic perturbation of each cell. Since CyTOF allows for detection of up to 45 distinct conjugated antibodies, numerous phenotypic targets can be matched to these Pro-Codes. This system has been implemented in breast cancer 4T1 cells to screen for positive and negative regulators of breast cancer immune evasion. This study has revealed that knockout of two interferon-stimulated genes, *Psmb8* and *Rtp4*, contribute to cancer cell resistance to T cell killing and that *Socs1* negatively regulates PD-L1 expression (120) (**Figure 1E**). Overall, ECCITE-seq, Perturb-CITE-seq, and CyTOF proteomic screening methods have demonstrated their potential in cancer cell line screens to reveal tumor immune-related mechanisms. However, additional studies are needed to apply these techniques for screening of immune cell populations.

## Coupling CRISPR screens to optical readouts

Pooled CRISPR screens can additionally be linked to single-cell optical phenotypes in fixed and live cells (**Table 1**). Initial work done by Feldman et al. (121, 127) details the use of barcoded single gRNA libraries as well as non-barcoded CROP-seq libraries for this purpose (**Figure 2A**). Following a pooled CRISPR screen, immunostaining and microscopy is employed to delineate single cells in a region of interest prior to *in situ* amplification and sequencing. This method can even detect multiple perturbations per cell for potential genetic interaction studies. Optical pooled screening has been applied to multiple human cancer cell lines and successfully screened human cervical cancer (HeLa) cells for NF-κB regulators in response to IL-1β or TNFα stimulation. To do this, p65 nuclear localization was used as an optical readout. In this study, *MED12* and *MED24* were found to be previously unknown negative regulators of p65 translocation and NF-κB activation (121). Therefore, this approach can uncover regulators controlling tumor cell responses to immune stimuli and may be applied to additional cell types in the future.

Pooled optical screens show promise for *in vivo* settings as well. Induction of CRISPR perturbations *in vivo* followed by imaging and *in situ* gRNA sequencing of extracted tissues allows for characterization of native spatial phenotypes, such as the tumor microenvironment (TME). Moreover, additional implementation of spatial transcriptomics can link genetic alterations with both optical and transcriptomic data. This approach has been recently exemplified by the Perturb-map system (22) (**Figure 2A**) that specifically uses Pro-Codes (120) to detect each perturbation and has been used to screen lung adenocarcinoma cells *in vivo* with a pooled library of known cytokine signaling regulators. Perturb-map has enabled visualization of immune cell infiltration and identification of molecular signatures in extracted lung tumors in response to specific perturbations (22). Notably, loss of TGFβ receptor (TGFβR2) in tumor lesions led to T cell exclusion and enrichment of a TGFβ-activated lung fibroblast signature, indicating stroma remodeling and a more immunosuppressive TME (22). Thus, Perturb-map is an exciting new avenue to uncover spatial regulators of the native TME.

## Analysis of high-content CRISPR screens

When conducting a conventional pooled CRISPR screen, quality control evaluations, including determination of the gRNA coverage and correlation between biological replicates are essential (128, 129). Inclusion and evaluation of negative non-target control guides can assist in limiting false positive discovery. To determine which genes are most relevant to the screened phenotype, numerous analysis tools have been developed (130, 131) including MAGeCK (132), HiTSelect (133), and CasTLE (134). Broadly, these tools evaluate the enrichment or significance of each sgRNA to deduce gene level analyses and rankings.

The primary readout of a high-content pooled CRISPR screen is sequencing data containing barcode or gRNA counts, which can then be used to detect and match each perturbation to a corresponding cell. Furthermore, transcriptome data is generated for scRNA-seq-based screens, microscopy data for optical screens, or cytometry data for CYTOF proteomic screens. Many bioinformatic methods have been developed to analyze scRNA-seq-coupled CRISPR screen data, such as FBA, LRICA, MELD, MIMOSCA, MILO, Mixscape, MUSIC, Normalisr, SCEPTRE, and scMAGeCK (118, 135–141).

The initial step to analyzing a pooled scRNA-seq CRISPR screen is processing the raw sequencing data. This can be done by using standard scRNA-seq analysis tools, or tools tailored for scRNA-seq-CRISPR screens such as FBA and scMAGeCK (135, 141) utilizing the barcode or gRNA sequences to demultiplex cells. Multiple analysis pipelines have been developed including aggregating cells based on gRNA expression followed by differential gene analysis of transcriptome profiles (109), or assignment of transcriptome signatures to each gRNA perturbation using principal component analysis (19). Low-rank independent component analysis (LRICA) can also distinguish components with distinct genetic perturbations (51). Conversely, unsupervised clustering can instead first group cells based on transcriptome profile, followed by analysis of enriched gRNA within each cluster (16, 108, 113). The effect of a given perturbation can then be calculated by comparing the average transcriptome profile of perturbed to control cells (108). A more specific analysis tool, MIMOSCA (Multi-Input-Multi-Output-Single-Cell-Analysis), was additionally created for analysis of Perturb-seq data (54). Furthermore, perturbation modules and affected gene programs can be visualized using heat maps (54).

More recently, defined packages have been created to analyze scCRISPR screen data including FBA, MELD, MILO, Mixscape, MUSIC, Normalisr, SCEPTRE, and scMAGeCK (118, 135–141). These tools include additional CRISPR-specific quality control steps, such as removing unperturbed cells or cells with invalid edits (138–140), filtering a minimum number of cells per perturbation (138), or detecting off-target effects (139). A feature barcoding analysis (FBA) package can be used to process and cluster cells based on their perturbations (135). Manifold learning algorithms and dimension reduction algorithms that estimate and visualize the relative likelihood ratios of perturbations influencing phenotypes have also been developed (136, 142). Furthermore, the MELD algorithm uses manifold learning to generate relative likelihood scores that may then be used for vertex frequency clustering (VFC) of perturbed cells (136). Finally, differential expression analysis can be performed on clusters or neighborhoods to obtain perturbation signatures.

Packages that have been validated with CRISPRi, such as SCEPTRE, Normalisr, and scMAGeCK, can additionally detect gene enhancer links and regulatory networks (139–141). scMAGeCK’s linear regression-based method, scMAGeCK-LR, is capable of simultaneously analyzing the expression of all genes in a cell to deduce networks, including cells possessing multiple perturbations. Meanwhile, the Robust Rank Aggregation (RRA) component, scMAGeCK-RRA, identifies regulatory relationships by ranking cells based on expression of a certain gene of interest, which is then linked to the cell’s corresponding perturbation (141). Finally, specific perturbations may be ranked according to their effect on a cell’s phenotype using the MUSIC algorithm (138). This ranking may be according to an overall perturbation effect, a functional topic-specific effect, or a relationship between different perturbations. These emerging algorithms are promising tools to ease analysis of high-throughput single-cell CRISPR screens evaluating tumor immunology in the future.

## Discussion and conclusion

Elucidation of gene function is key to the discovery of prospective therapeutic targets in many disease contexts. The CRISPR-Cas9 system has become a central tool in this search by enabling genetic screens to be easily performed at the genome scale. Recent studies have effectively coupled CRISPR screens to content-rich phenotypes using transcriptomic, epigenetic, proteomic, and optical readouts. These systems can deconvolute genotypic phenotypic relationships and have successfully identified regulators of numerous contexts including T cell stimulation and exhaustion, ICI resistance, and cancer immune evasion.

In recent years, CRISPR technology has been extended to the clinic (143–145) (**Figure 2D**). Initial proof-of-concept clinical trials in advanced esophageal (NCT03081715), metastatic non-small cell lung (146), and advanced, refractory cancer (NCT03399448) (147) have demonstrated CRISPR-Cas9 editing of autologous CAR T cells prior to adoptive transfer to be safe and feasible. Ongoing Phase I clinical trials have used CRISPR editing to remove disadvantageous genes from autologous CAR T cells, such as HPK1 (NCT04037566) or TGFβR (NCT04976218), to improve the T cell antitumor immune responses. CRISPR-Cas9 editing has also been used in allogeneic CAR T cells to diminish immunogenicity through the deletion of β2 microglobulin as well as enable precise insertion of the CAR construct into the endogenous *TRAC* locus (NCT04035434, NCT04557436, NCT04637763, NCT04244656, NCT04502446). The PoKI-seq and ModPoKI-seq studies highlight the potential of high-content CRISPR screens to enhance CAR T function (32, 110). In the future, high-content CRISPR screens in immune cells can be used to identify genes controlling antitumor activity, such as activation or effector functions (**Figure 1**). These scientific advances can be rapidly translated into immune cell therapeutics with the potential to improve cancer care (**Figure 2D**).

Despite its influential application in gene discovery, there remain limitations to CRISPR screening. While *in vitro* genome wide screens can be performed in immune cells and have discovered novel regulators of T cell activation and proliferation (16, 90, 91), these readouts only partially approximate T cell biology. Pooled *in vivo* T cell screens can offer a more holistic evaluation of T cell function, but the low number of tumor-infiltrating lymphocytes can contribute to dropouts and false negatives (99). Additionally, many analysis tools for high-content CRISPR screens have yet to be extensively implemented, warranting future validation in the context of tumor immunology. Furthermore, although pooled CRISPR screens have been used to elucidate intercellular regulators governing cell-to-cell interactions (107), screens that focus on intercellular regulators remain largely unexplored. In the future, additional pooled CRISPR screens which focus on intercellular regulators are needed to better dissect the complexities of interactions within the tumor microenvironment.

In conclusion, high-content screens are likely to become a core approach for the future of CRISPR. The application of this technology to the tumor immunology field has the potential to reveal genes that regulate antitumor function. These genes may be therapeutically targeted in a clinical setting to improve patient outcome.

## Author contributions

EH and MG conceptualized this review and drafted the original manuscript. AP, KJ, AT, JJ, LJ, and AH contributed to the writing and critical revision. All authors have approved the final submission.

## Funding

Funding was provided to EH by the Immunology Training Program. Funding was provided to KJ by the Pharmacological Sciences Training Program (PSTP) T32 Training Grant (GM007767), the Rackham Merit Fellowship, and the Rackham Graduate School Research Grant. Funding was provided to AP by NIAID Training Grant T32 (AI007413). Funding was provided to MG by the Lung Precision Oncology Program (VA 150CU000182), LUNGevity, Veterans Affairs (I01 BX005267), Melanoma Research Alliance (MRA689853), NCI (CA252010), and the Breast Cancer Research Foundation.

## Acknowledgments

Figures were created with BioRender.com.

## Conflict of interest

The authors declare that the research was conducted in the absence of any commercial or financial relationships that could be construed as a potential conflict of interest.

## Publisher’s note

All claims expressed in this article are solely those of the authors and do not necessarily represent those of their affiliated organizations, or those of the publisher, the editors and the reviewers. Any product that may be evaluated in this article, or claim that may be made by its manufacturer, is not guaranteed or endorsed by the publisher.
